# Barriers of the CNS transfer rate dynamics in patients with vascular cognitive impairment and dementia

**DOI:** 10.3389/fnagi.2024.1462302

**Published:** 2024-09-25

**Authors:** Saeid Taheri, Jill Prestopnik, Gary A. Rosenberg

**Affiliations:** ^1^Department of Pharmaceutical Sciences, University of South Florida, Tampa, FL, United States; ^2^Center for Functional and Molecular Imaging, University of South Florida (USF) Heart Institute, Tampa, FL, United States; ^3^Center for Memory and Aging, Albuquerque, NM, United States; ^4^Department of Neurology, Health Sciences Center, University of New Mexico, Albuquerque, NM, United States

**Keywords:** blood-brain barrier, vascular cognitive impairment and dementia (VCID), dynamic contrast-enhanced MRI (DCE-MRI), blood-cerebrospinal fluid barrier, MR spectroscopy, inflammation, cluster analysis

## Abstract

**Background:**

Advances in *in vivo* MRI techniques enable cerebral barrier transfer rates (K_*trans*_) measurement in patients with vascular cognitive impairment and dementia (VCID). However, a consensus has not been reached on the dynamic contribution and importance of cerebral barrier abnormalities to the differential diagnosis of dementia subtypes. Our goal was to investigate the dynamics of blood-brain barrier (BBB) and blood-CSF barrier (BCSFB) K_*trans*_ in patients with VCID longitudinally and determine the effect of aging.

**Methods:**

We studied subjects at two time points over two years; they were 65.5 years of age (SD = 15.94, M/F = 24/14) at the first visit. We studied 38 patients, 18 of whom had two visits. We calculated the BBB and BCSFB K_*trans*_ with dynamic contrast-enhanced T1 MR, and we used ^1^H-MR spectroscopy to measure N-acetylaspartate (NAA) levels in the white matter as a marker of injury. In addition, we measured CSF levels of active-matrix metalloproteinase-3 (MMP3) as an inflammatory biomarker to aid in patient clustering.

**Results:**

Longitudinal BBB measurements revealed variable dynamic behavior: after two years, the BBB K_*trans*_ increased in 55% of patients and decreased in the remaining 45% unpredictably. We did not find a significant linear model of BBB K_*trans*_ versus age for VCID. For healthy controls, the model was K_*trans*_ = 0.0014 + 0.0002 × age, which was significant (*p* = 0.046). VCID patients showed a reduction in BCSFB K_*trans*_ compared to healthy controls (*p* = 0.01). Combining NAA, CSF MMP3, and K_*trans*_ in a clustering analysis separated patients into groups.

**Conclusion:**

These results suggest that BBB K_*trans*_ in VCID is dynamic and BCSFB K_*trans*_ reduced by age. By combining inflammatory biomarkers with BBB K_*trans*_ data, it is possible to separate VCID patients into distinct groups with different underlying pathologies.

## 1 Background

The prevalence of dementia is increasing with aging populations worldwide. The most common form of dementia is Alzheimer’s disease (AD), but recent studies indicate a prominent role in dementia for vascular disease (Toledo et al., 2013; Schneider et al., 2007). Vascular cognitive impairment and dementia (VCID) is a heterogeneous group of cognitive disorders that share presumed cerebrovascular diseases. When both abnormal proteins and damaged blood vessels are present, an inflammatory response occurs that induces extracellular proteases that disrupt the blood-brain barrier (BBB) by attacking the basal lamina and loosening blood vessel tight junctions (Vercellino et al., 2008). Numerous cerebrovascular diseases affect the widely studied cerebral barriers, the BBB, and the blood-CSF barrier (BCSFB). In particular, BBB function alterations have been reported in both focal and diffuse abnormalities observed in the brain with inflammation, such as multiple sclerosis (Vos et al., 2005) and VCID (Taheri et al., 2011a). Although it is accepted that alterations in these barriers limit their protection against inflammatory mediators and escalate inflammation, the impact of injury to the BBB and CSFB is not fully understood. It is now possible to determine the extent of injury by assessing barrier dynamics, cerebrospinal fluid (CSF) circulation, and inflammation, thanks to advances in quantitative *in vivo* imaging and the ability to measure the impact of inflammation on barriers noninvasively.

Neurovascular dysfunction and vulnerability to cerebrovascular diseases are associated with arterial aging, as evidenced by increased arterial stiffness and reduced vascular reactivity (Farrall and Wardlaw, 2009). For example, the permeability of the BBB increases with age (Ford et al., 2022; Andjelkovic et al., 2023; Senatorov et al., 2019; Montagne et al., 2015), and CSF production and circulation decrease with age (Rubenstein, 1998; Chiu et al., 2012). In addition, aging may exacerbate BBB and BCSFB disruptions caused by pathological conditions (Ueno et al., 2016). In aging-related diseases such as VCID, increased BBB transfer rate (K_*trans*_) is believed to be a predictor of injury progression (Hussain et al., 2021).

Therefore, we can use K_*trans*_ as a measure of barrier health and to categorize VCID patients according to the severity of K_*trans*_. In our studies, we showed that various biomarkers may differentially reflect VCID disease severity (Taheri et al., 2011b; Taheri et al., 2011a; Gasparovic et al., 2013; Candelario-Jalil et al., 2011). In this report, we hypothesized that a multimodal statistical classification method could differentiate VCID patients based on their most likely pathological contributors. An investigation of biomarkers collected at the University of New Mexico was also conducted to test this hypothesis. Various methods of statistically clustering subjects based on biomedical data have been used to classify patients (Jain et al., 1999). Model-based clustering is a technique for estimating group membership based on parametric mixture models (Fraley and Raftery, 2002; Browne and McNicholas, 2012). In the model-based clustering literature, the finite Gaussian mixture model is most commonly used (McNicholas and Murphy, 2010). We then used a multi-parametric mixture modeling approach (Bertoletti et al., 2015; Grun and Leisch, 2008) to blindly cluster VCID patients into related subgroups.

The purpose of this study was to determine the impact of aging on BBB K_*trans*_ and BCSFB K_*trans*_ in a group of VCID patients and compare with healthy controls. Our main hypothesis was that when cerebrovascular injury develops in VCID, the BBB K_*trans*_ increases and the BCSFB K_*trans*_ decreases. To test this hypothesis, we used DCE-MRI to quantify BBB K_*trans*_ (Taheri et al., 2011b) and BCSFB K_*trans*_ (Evans et al., 2020). In addition, we used N-acetylaspartate (NAA) to indicate white matter injury and MMP3 to indicate inflammation. Using the BBB K_*trans*_ value with other biomarkers, we examined whether it is possible to differentiate patients with VCID according to the severity of the disease. As a contribution to the literature, this study highlights the dynamic nature of the BBB K_*trans*_ in patients with VCID. Furthermore, given the dynamic changes in the BBB K_*trans*_, we propose the importance of a multimodal approach in VCID classification, using a combination of biomarkers.

## 2 Materials and methods

### 2.1 Subjects

Patients were recruited from the Center for Memory and Aging Clinic at the University of New Mexico Hospital, and from the Memory Clinic at the Albuquerque Veterans Administration Hospital. The experiments were strictly randomized and blinded. Statistical planning assumed an α-error of 5% and a β-error of 20%. Participants included in this analysis included 18 VCID patients and 15 healthy controls (HC). The HCs had an average age of 51 years (SD = 19, M/F = 10/7), whereas the patients had an average age of 65.5 years (SD = 15.94, M/F = 24/14). Among the 38 patients who participated in the study, we acquired complete data for three biomarkers: NAA compounds, mean BBB K_*trans*_, and active CSF MMP3. Of these patients, 18 were able to participate in a BBB K_*trans*_ follow-up study. The average age of the follow-up patients enrolled in this study was 62 years (SD = 20.36, M/F = 12/6). The demographic data of patients and controls who participated in this study are summarized in Table 1.

**TABLE 1:** Clinical, demographic and neuropathological characteristics of patients and controls participated in the study.

	VCID 1^st^ visit		VCID 2^nd^ visit		Controls one visit	
	*N*	*IQR*	*N*	*IQR*	*N*	*IQR*
Total	38		18		15	
Age *(median)*	65.5	20–87	67	22–89	61	22–75
**Sex**						
Male	24	63.2%	12	66.6%	10	58.8%
Female	14	36.8%	6	33.3%	7	41.2%
Stroke	17	45%	8	44.4%	0	0%
MMSE *(average)*	36	27	36			—
Missing	2					
Hypertension	18	43%	12	66.6%	0	0%
Hypercholesterolemia	23	60%	10	55.5%	0	0%
Sleep Apnea	10	26%	6	33.3%	0	0%
Missing	9					

IQR, Interquartile range.

### 2.2 Specific inclusion/exclusion criteria

Patients must meet all the following criteria for admission to this study: (1) signed, written informed consent, (2) male or nonpregnant, nonlactating female patients, and (3) had cognitive impairment and had ischemic vascular abnormalities on MRI. The questionnaire included patients with a clinical dementia rating (CDR) scale of 0, 0.5, or 1.0. Patients who met any of the following criteria were excluded from the study: (1) had a history of malignancy (except for basal cell skin carcinoma, for which the patient was eligible only if disease-free for 5 years or more), (2) had any disability acquired from trauma or another illness that, in the opinion of the investigator, could interfere with the evaluation of disability due to AD or mild cognitive impairment (MCI), (3) were unable to undergo MRI with gadolinium administration, (4) had untreated major depressive disorder (MDD), (5) had epileptic seizures that were not adequately controlled by treatment, and (6) had suicidal ideation.

### 2.3 Data collection

The MRI data were acquired with a 1.5 Tesla Siemens Sonata scanner with a standard eight-channel array head coil (Siemens AG, Erlangen, Germany). As part of the MR protocol, we performed structural imaging, dynamic contrast-enhanced MRI (DCE-MRI) and ^1^H-MR magnetic resonance spectroscopy (^1^H-MRS). In a DCE-MRI procedure, a series of T1 map images were acquired before and after an optimized dose of Gadolinium diethylene triamine pentaacetic acid (Gd-DTPA, MW = 938 Da; Bayer Healthcare) was injected as a contrast agent (Taheri et al., 2016).

Data acquisition for calculating BBB transfer rates was based on a series of 8 T1 maps acquired with a fast T1 mapping sequence before and after the Gd-DTPA injection. One T1 map was acquired before Gd-DTPA injection, and the rest were acquired post-injection, resulting in a 2D time series dataset of contrast-enhanced MR images. The T1 mapping sequence used was TAPIR (T1-mapping with partial inversion recovery) (Shah et al., 2001; Neeb et al., 2006). Data were acquired with six 5-mm slices in the axial plane centered above the lateral ventricles and parallel to the anterior-posterior commissure axis (TR/TE/TI = 13 ms/2 ms/30 ms, flip angle = 25 degrees, FOV = 220 mm × 220 mm, slice thickness = 5.0 mm, slice gap = 5 mm, number of slices = 6, number of averages = 1, matrix size = 128 × 128, receiver bandwidth = 50 kHz). This parameter selection results in an in-plane resolution of 0.582 voxels/mm and a sampling interval of approximately three minutes. ^1^H-MRS was used to investigate the levels of both total creatine (Cr) and NAA in the white matter region above the lateral ventricles. ^1^H-MRS was performed with a phase-encoded version of a point resolved spectroscopy sequence (PRESS) with FOV = 220 mm × 220 mm and slice thickness = 15 mm. The nominal voxel size was 6.88 × 6.88 × 15 mm^3^. For more details on MRS data acquisition, see a previous paper (Gasparovic et al., 2013). We ensured that all follow-up imaging sessions were conducted in the same manner, including anatomical locations and MR sequence parameters.

The levels of active MMP3 in the CSF were measured with a fluorometric activity assay and were used to stratify patients according to patient clustering. A description of CSF MMP data collection and processing has been provided previously and will be briefly described (Candelario-Jalil et al., 2011). The activity of MMP-3 (stromelysin-1) in CSF and plasma was measured fluorometrically using a 5-FAM/QXL520 fluorescence resonance energy transfer peptide. With the intact fluorescence resonance energy transfer peptide (5-FAM-Arg-Pro-Lys-Pro-Val-Glu-Nva-Trp-Arg-Lys[QXL520]-NH_2_), the fluorescence of 5-FAM (5-carboxyfluorescein) is quenched by QXL520. Upon cleavage into 2 separate fragments by MMP3, the fluorescence of 5-FAM is recovered and can be monitored at excitation/emission wavelengths of 490/520 nm. This peptide has been shown to be cleaved by only MMP-3 and MMP-12 but not by other MMPs (Jain et al., 1999).

### 2.4 BBB transfer rate (K_*trans*_) calculations

The details of the K_*trans*_ calculations were described previously (Taheri et al., 2011a; Taheri et al., 2011b). In brief, K_*trans*_ was calculated using Patlak compartmental analysis on dynamic contrast-enhanced T1 maps with Gd-DTPA as a contrast agent. Color-coded K_*trans*_ maps were subsequently generated. The mean BBB K_*trans*_ for WM was calculated from voxel-by-voxel data with a voxel size of 0.34 × 0.34 × 5 mm^3^ for six consecutive slices with a 5 mm thickness starting from the top of the brain. We used the same codes and software to process the follow-up data.

Three methods were used to interpret elevated BBB K_*trans*_ at the voxel level: (1) mean elevated BBB K_*trans*_, which includes voxels with values higher than the average normal BBB K_*trans*_ value in healthy control brain (Taheri et al., 2011b); (2) mean elevated BBB K_*trans*_ voxel values in WM; and (3) total elevated BBB K_*trans*_ in WM, which represents the sum of all voxel values in WM. Notably, the difference between the mean elevated BBB K_*trans*_ and the total elevated BBB K_*trans*_ represents the BBB K_*trans*_ intensity.

To measure the choroid plexus (CP) transfer rate, a bolus of contrast agent (Gd-DTPA) was injected intravenously, followed by 25 min of sampling within a region of interest (ROI) placed in one of the lateral ventricles excluding the choroid plexus. A rectangular ROI of 10 × 20 voxels was placed within the ventricular area without covering any tissues. The concentration of Gd-DTPA ([Gd-DTPA]) in the ventricular area was determined using the average of the acquired time series of T1 images in ROI. For quantitative measurements of [Gd-DTPA], a quarter dose of 0.1 mmol/kg allowed us to use a linear approximation between T1 and [Gd-DTPA]. Based on the widely accepted constant-relaxivity relationship between T1 and [Gd-DTPA], [Gd-DTPA] was estimated in the ROI of the T1 map images of the ventricles using the constant-relaxivity relationship. 1/T1 = r1*[Gd-DTPA]. Where r1 is the relaxivity (mM^–1^s^–1^; which is reported to be 4.79 for 1.5T) and [Gd-DTPA] is in mmol. The reduction in the T1 value in the ventricular cavity reflects the rate at which [Gd-DTPA] increases in the ventricular cavity through the CP. As Gd-DTPA can easily pass through CP, it is suitable for measuring CP transfer rate. Therefore, we quantified the rate of CP transfer through the BCSFB by measuring the change in [Gd-DTPA]. Of note is that this method enables us to measure the transfer of substances whose molecular weight is lower than or equal to that of Gd-DTPA. The CP transfer rate was then calculated using the same procedure proposed for calculating the BBB K_*trans*_. In this study, we examined the CP transfer rate of 12 healthy controls and compared it to that of 12 patients with VCID.

### 2.5 Statistical analysis

Quantitative data was analyzed using the “R” platform (version 4.3.2, R Development Core Team, 2023). All the datasets were tested for normality using the Shapiro–Wilk test, and a subsequent unpaired *t*-test or Mann–Whitney test was applied based on the presence of a parametric or nonparametric distribution, respectively. We performed the Dixons Q test in R to exclude outlier in the analysis. Parametric statistical comparisons between the datasets were made based on the representation of the mean ± standard deviation (SD). Student’s *t*-test (for nonparametric data) was used for statistical analyses. Statistical analyses between groups were performed using two-way repeated measurements and analysis of variance (ANOVA) with Tukey’s *post-hoc* tests for multiple comparisons. A linear regression model (*lm* in R) was used to fit a line to the data for comparison. R^2^ was used as a goodness-of-fit. We considered a *P*-value < 0.05 as indicating statistical significance.

### 2.6 Statistical classification method

A multi-parametric model-based method was used to blindly classify VCID data using K_*trans*_, NAA, and active MMP3. A mixed modeling approach assumes data from different sources, and each source is modeled separately. We assumed that the mixture of VCID patients consisted of K clusters, with a specific distribution for each cluster. Patient v of VCID is assigned a probability Pvk to cluster k, where each cluster follows a parametric distribution.

The mixture distribution is given by the weighted sum of the K clusters. The n-dimensional vector x = (x_1_, …, x_*n*_)*^T^* contains the values of n variables measured for each of the VCID patients. The mixture density of variable x for patient j, x_*j*_ is


$$f\,\left(x_{j},\varphi \right)=\sum_{k=1}^{K}\pi_{k}\,f_{k}\,\left(x_{k\,j},\theta_{k}\right)\,j=1,\cdots ,V$$

Where φ is the vector of all unknown parameters of the model, *f*_*k*_ is the specific density function of cluster k, and π_*k*_ is the weight of the *k*th cluster that can be considered as *a prior* probability for an observation to come from this cluster. Model parameters were estimated by the expectation maximization (EM) algorithm, where the E-step was used to determine a-posteriori probabilities and the M-step was used to maximize the likelihood function.

This study was conducted using the flexible mixture analysis package (flexmix) (Grun and Leisch, 2008; Lo and Gottardo, 2012) in the R environment provided by the R Core Team (R Development Core Team, 2023). The number of clusters was optimized using the Bayesian information criterion (BIC) and the integrated completed likelihood (ICL). In mixture models, the ICL criteria are commonly used for clustering data by automatically selecting the number of clusters (Bertoletti et al., 2015; Vrieze, 2012). After selecting the number of clusters, we classified VCID patients into different clusters based on their entity values. Next, we provided estimated values for entities in each cluster. These values could be used to assign VCID patients to different clusters.

## 3 Results

**FIGURE 1:**
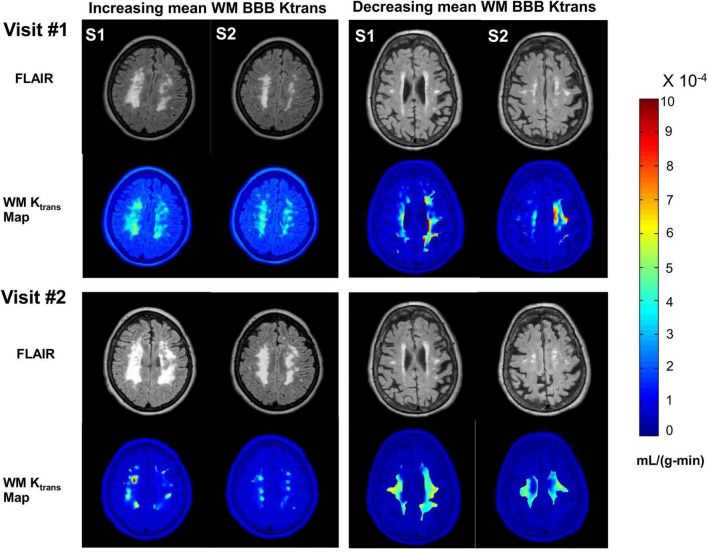
An illustration of BBB Ktrans dynamics in two patients for two years. Right panel shows a patient with increasing mean WM BBB Ktrans and the left panel shows a patient with decreasing mean WM BBB Ktrans within two years. In this figure, two consecutive axial slices of the brain of a patient with VCID acquired by structural- and DCE-MRI two years apart are depicted. In the upper rows of each panel, FLAIR anatomical images of the two slices are displayed. Each panel displays K_trans_ maps overlaid on FLAIR anatomical images representing white matter in the lower rows. The S1 and S2 slices were matched between the two visits. A K_trans_ map was created by color-coding each pixel’s K_trans_. The color bar on the right indicates K_trans_ values in mL/(g-min). Based on FLAIR MR imaging, the white matter injury appears to be progressing in the left panel, whereas there is no significant change in the right panel. Within two years, the mean WM BBB damage for the patient depicted in the left panel has become more severe and extensive.

BBB damage in patients with VCID is dynamic. WM BBB transfer rates (K_*trans*_) of two patients with VCID are shown in Figure 1. FLAIR anatomical and corresponding WM BBB K_*trans*_ maps of two consecutive axial slices of two patients with VCID acquired by anatomical and DCE-MRI two years apart are shown in this figure. In the left panel of this figure, mean WM BBB leakage increased as it is visible in the bottom row compared with the top row of K_*trans*_ maps. For this patient, the hyperintensity area on the FLAIR image also increased. By contrast, the left panel depicts a patient with a decrease in mean WM K_*trans*_ within two years. For this patient, the hyperintensity in FLAIR did not increase.

Longitudinal BBB K_*trans*_ measurements do not reveal a pattern for VCID patients. Table 2 shows the longitudinal mean elevated BBB K_*trans*_ for the sampled brain parenchyma of VCID patients at two consecutive times. There is no conclusive pattern in BBB K_*trans*_ progression, as K_*trans*_ increases in 55% of patients and decreases in the remaining 45% in an unpredictable manner. Table 2 shows the mean BBB K_*trans*_ for patients with VCID white matter (WM) was calculated. Similar to K_*trans*_ in the entire brain parenchyma, this figure illustrates that there is no clear pattern for K_*trans*_ changes in WM. In half of the patients, the mean WM K_*trans*_ increases, while in the other half, it decreases.

**TABLE 2:** BBB K_trans_ dynamics in patients with VCID in a two-year follow-up study.

		BBB K_trans_ in brain parenchyma (10^–4^ mL/g-min)			BBB K_trans_ in WM (10^–4^ mL/g-min)		
**Patient**	**Age**	**1st visit**	**2nd visit**	**Δ K_trans_**	**1st visit**	**2nd visit**	**Δ K_trans_**
p20	48	2.51	1.10	−1.407	0.57	0.57	0.000
p18	50	1.78	1.79	0.010	0.57	1.24	−0.665
p46	51	1.09	1.09	0.001	0.49	0.49	0.000
p12	56	2.04	1.87	−0.169	0.83	2.93	−2.086
p37	60	1.91	1.28	−0.630	0.54	0.80	−0.252
p36	62	0.98	3.32	2.335	0.25	0.75	−0.495
p25	64	2.01	2.20	0.185	1.77	3.28	−1.506
p38	66	3.39	1.38	−2.004	1.77	0.50	1.263
p39	66	1.38	0.96	−0.384	0.57	0.39	0.181
p19	66	2.41	1.17	−1.240	0.82	0.38	0.435
p44	67	4.20	1.23	−2.970	1.70	0.54	1.160
p35	71	2.81	2.55	−0.260	1.21	0.76	0.449
p21	72	0.89	2.36	1.458	0.29	0.84	−0.554
P04	72	0.92	1.22	0.294	0.71	0.41	0.301
p34	76	1.83	1.67	−0.155	0.84	0.57	0.266
p10	81	5.16	3.82	−1.340	1.18	2.29	−1.109
p40	82	1.59	4.85	3.259	1.16	0.53	0.622
p41	87	3.00	1.37	−1.630	1.19	0.74	0.450

The BBB K_trans_ of 18 VCID patient brains were calculated at two points over a two-year period. In middle two columns, the final value of BBB K_trans_ was calculated by averaging voxelwise K_trans_ values in the sampled brain parenchyma with the exclusion of the CSF compartment. As is evident from this Table, there is no clear increasing pattern for K_trans_ within two years in VCID brains. The mean BBB K_trans_ for patients with VCID white matter (WM) was also calculated. Similar to K_trans_ in the whole brain, this Table also illustrates that there is no clear pattern for K_trans_ changes in WM. ΔK_trans_ shows the difference between 1st visit K_trans_ and the 2nd visit K_trans_.

Supplementary Figure 1A illustrates the total elevated BBB K_*trans*_ dynamics in WM for each patient. The total elevated BBB K_*trans*_ is another measure that can be used to separate cases where there is a small area with high BBB K_*trans*_ versus a larger area with small BBB K_*trans*_. Even though more than 50% of patients showed an increase in the WM BBB K_*trans*_, we were unable to identify a longitudinal pattern in the WM BBB K_*trans*_. We sampled the superior sagittal sinus area as the arterial input function (AIF). To test the rigor of the BBB K_*trans*_ calculation, we compared the AIF at both visits. There was no statistically significant difference in the AIF of VCID patients between the first and second visit (Supplementary Figure 1B).

**FIGURE 2:**
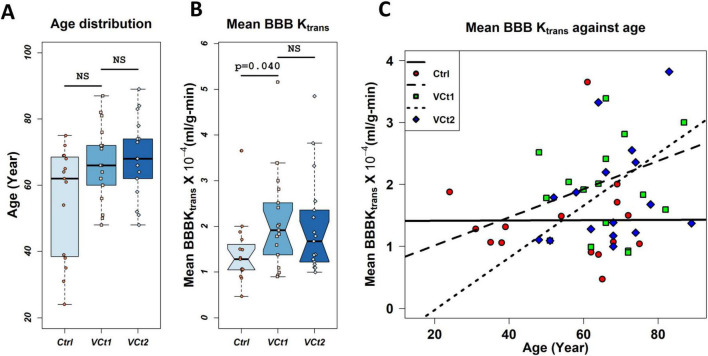
Plots of BBB transfer rate (K_trans_) against age. Panel **(A)** compares the age distribution of VCID patients and healthy controls used in this study. The age distribution of these three groups did not differ significantly (NS) [*p* = 0.062, N controls (Ctrl) = 15, N VCID at first time (VCt1) = 18, N VCID at second time (VCt2) = 18]. Panel **(B)** represents a comparison of the average BBB K_trans_ for VCID patients at two times and healthy controls in the whole brain parenchyma with the exclusion of the CSF compartment. There is a statistically significant difference between the mean BBB K_trans_ of controls and VCt1 (*p* = 0.040). However, we did not find any statistically significant difference between mean BBB K_trans_ of VCID at two times. Mean BBB K_trans_ was calculated voxel-wise with a voxel size of 0.34 × 0.34 × 5 mm^3^ (sampled with six consecutive slices with a 5 mm thickness). *N* = 15 controls and 18 VCID subjects were used in this analysis. Panel **(C)** represents the distribution of mean BBB K_trans_ in healthy controls and VCID subjects at two times, respectively, against age. A linear regression model was used to investigate the impact of age on these two groups’ BBB K_trans_. We did not find a statistically significant linear model of BBB Ktrans versus age for VCID. The model for controls was K_trans_ = 0.0014 + 0.0002 × age which was statistically significant (*p* = 0.046).

Older VCID patients had a greater average BBB K_*trans*_. We observed a greater slope for VCID patients than for control patients when we fit the age distribution of the average BBB K_*trans*_ with linear regression. In Figure 2A, the age distributions of VCID patients and healthy control subjects are shown. The age distribution of these three groups did not differ significantly (NS) [*p* = 0.062, N controls = 15, N VCID at first time (VCt1) = 18, and N VCID at second time (VCt2) = 18]. Figure 2B represents a comparison of the average BBB K_*trans*_ for VCID patients at two times and healthy controls in the whole brain parenchyma with the exclusion of the CSF compartment. There is a statistically significant difference between the mean BBB K_*trans*_ of controls and VCt1 (*p* = 0.033). However, we did not find any statistically significant difference between mean BBB K_*trans*_ of VCID at two times. Figure 2C shows the distribution of the mean BBB K_*trans*_ in the whole brain parenchyma, excluding the CSF compartment in healthy controls and VCID patients as a function of age. To find a meaningful relationship between the age of patients and BBB K_*trans*_, we fitted a linear regression model to both VCID and control data. We did not find a statistically significant linear model of BBB K_*trans*_ versus age for VCID (for both t1 and t2). For controls, the model was K_*trans*_ = 0.0014 + 0.0002 × age, which was statistically significant (*p* = 0.046). This equation indicates a slight increase in BBB K_*trans*_ with age in healthy controls.

**FIGURE 3:**
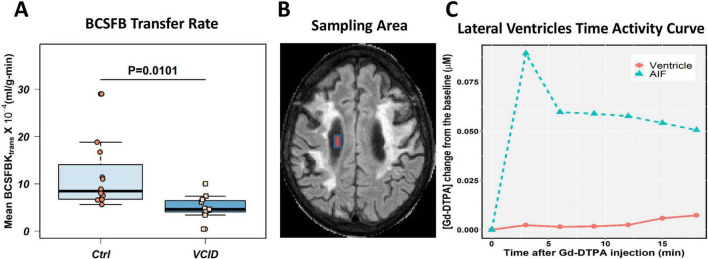
Choroid plexus transfer rates in VCID patients are lower than those in healthy controls. The blood cerebrospinal fluid barrier (BCSFB) transfer rates were calculated for twelve healthy controls and twelve VCID age- and sex-matched subjects whose DCE-MRI data covered the ventricular spaces. Patlak compartmental modeling techniques were used to calculate the BCSFB transfer rate. **(A)** Average BCSFB transfer rate in VCID is significantly reduced when statistically compared with healthy controls, with a *p*-value of 0.0101. Panel **(B)** illustrates the sampling area in the lateral ventricular area for BCSFB transfer rate calculation. An illustration of Gd-DTPA concentration ([Gd-DTPA]) time activity curve (TAC) in the ventricular area can be found in Panel **(C)**. This curve shows delayed leakage of Gd-DTPA into the ventricular area in a VCID patient in comparison to sampled arterial input function (AIF) that shows Gd-DTPA wash-in and wash-out periods.

The BCSFB transfer rate is reduced in VCID patients. We also compared the transfer of contrast agent from blood to CSF between VCID patients and healthy controls by measuring the transfer rate of Gd-DTPA into the lateral ventricles. In patients with VCID, the percentage of Gd-DTPA that passed through BCSFB into the ventricular area was significantly lower than in healthy controls. Furthermore, we observed that Gd-DTPA accumulation in the ventricles occurs at a slower rate than the AIF. After using compartmental modeling to calculate K_*trans*_, we observed that the average CP transfer rate in VCID patients was significantly lower than that in healthy controls (*p*-value = 0.0101; Figure 3A). Figure 3B shows the sampling area of a lateral ventricle. A sample [Gd-DTPA] time-activity curve (TAC) in the lateral ventricle of a patient with VCID is shown in Figure 3C. The ventricular TAC of the VCID group showed delayed and gradual accumulation of Gd-DTPA compared to that of the control TAC group. This figure also compares the VCID ventricular TAC with Gd-DTPA TAC of the superior sagittal sinus which is used as an arterial input function (AIF) to calculate K_*trans*_. The Gd-DTPA TAC shows wash-in and wash-out periods of Gd-DTPA.

**FIGURE 4:**
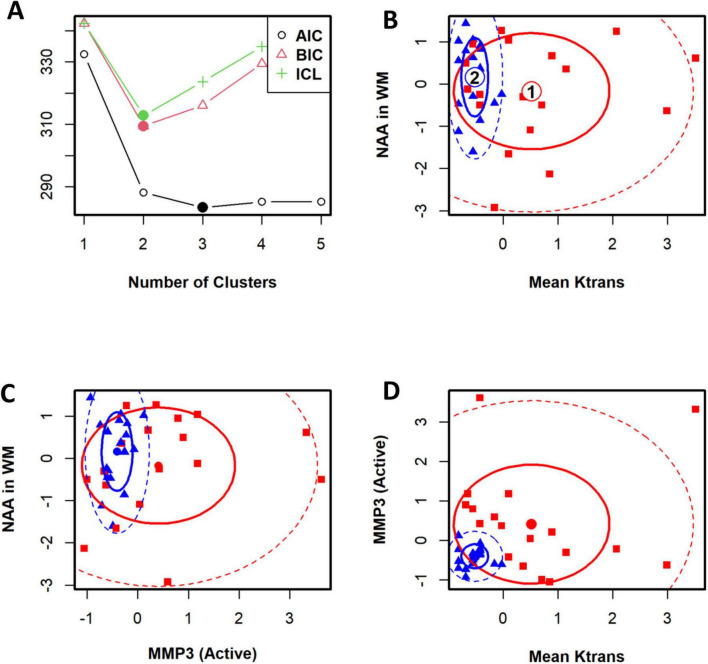
The cohort of patients with VCID has been classified statistically into two groups. To determine the optimal number of clusters for patients, an information criterion is used as a first step. Panel **(A)** illustrates the Bayesian information criterion and the integrated completed likelihood (ICL) optimized for two clusters. An optimal ICL was used to determine the number of clusters. Panels **(B–D)** illustrate ellipse plots for Gaussian mixtures fitted by *FLXMCmvnorm* at 50 and 95% confidence levels. This model identified two clusters. A flexible mixture analysis was used to cluster patients based on three parameters: mean K_trans_ in WM, NAA in WM, and active-MMP3. Flexible mixture analysis is a model-based mixture analysis method.

A statistical classification of VCID patients based on the BBB K_*trans*_. A panel of correlated parameters, such as lesion volume and NAA concentration in WM, did not cluster VCID patients. However, the use of a panel of biomarkers from different sources enables us to cluster patients with VCID successfully. Figure 4 illustrates the use of cerebral metabolites (NAAs) to stratify patients in conjunction with a parameter derived from known CSF biomarkers of inflammation (MMP3) and an imaging biomarker (BBB K_*trans*_). In this figure, we can see that the BBB K_*trans*_ value provides additional information on NAA and MMP3 levels. The figure illustrates the clustering power of a biomarker regarding the WM measurement area. In this study, the BBB K_*trans*_ was calculated within the WM areas of the covered brain volume. To cluster patients, we compared a panel of BBB K_*trans*_ values in WM with MMP3 to a panel of BBB K_*trans*_ values in WM without MMP3. According to this figure, WM BBB K_*trans*_ (including lesions areas), has greater clustering power if combined with other biomarkers.

In Figure 4, the top left panel depicts the Bayesian information criterion as well as an integrated completed likelihood (ICL) optimized for two clusters. Using the ICL, we could identify two statistically distinct groups of patients (Figure 4A). We identified two clusters using this model. In the remaining panels, ellipse plots are shown for Gaussian mixtures fitted with FLXMCmvnorm at 50 and 95% confidence levels (Figures 4B–D).

**FIGURE 5:**
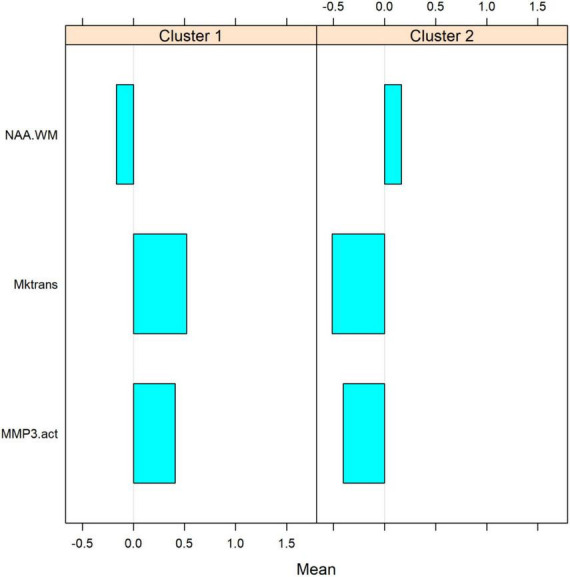
Estimated mean values of biomarkers within each cluster. The figure shows the normalized values of three biomarkers used for clustering VCIDs. As it is clear, each cluster has a different estimated value for these parameters. Based on these values, VCID patients could be assigned to one of these clusters. After using a statistical method to automatically cluster VCID patients into two groups, we compare biomarker values between the two clusters. VCID patients can be classified into two clusters using these metrics (which were estimated for selected biomarkers).

The metrics for VCID clustering were determined using the estimated mean values of the selected biomarkers in each cluster. After using a statistical method to cluster VCID patients into two groups automatically, we compared the entity values between the two clusters. This provides a metric for assigning VCID patients to a proper cluster. Figure 5 shows the normalized values of the three biomarkers that we used for VCID patient clustering. As is clear, each cluster has different estimated values for these biomarkers. We could classify VCID patients into one of two clusters by estimating metrics for the selected biomarkers.

## 4 Discussion

Our longitudinal study findings suggest that the *in vivo* quantified BBB transfer rate (K_trans_) is dynamic in patients with VCID. Even though we found that BBB K_trans_ was greater in older patients with VCID, we could not find a linear relationship between BBB K_trans_ and age. We could fit a linear model to the mean BBB K_trans_ with age in healthy controls. This highlights the difference between an individual longitudinal study and a mean study of BBB K_trans_ in VCID patients. On the other hand, BCSFB K_trans_ was reduced in this group of patients. Along with other elements, such as active MMP3 and NAA, the BBB K_trans_ contributes significantly to VCID classification. A model-based clustering method was used to cluster VCID patients into two clusters. The severity of the underlying pathology in VCID patients can be effectively stratified by using a combination of biomarkers, including the BBB K_trans_.

Several cerebral inflammatory diseases affect the BBB and BCSFB tight junctions (Abbott, 2000; Saul et al., 2020). BBB K_trans_ alterations have been reported in both focal and diffuse abnormalities in cerebral diseases, such as MS (Vos et al., 2005) and VCID (Taheri et al., 2011a). It has been shown that BCSFB disruption occurs in a variety of cerebral conditions, including AD (Brkic et al., 2015) and amyotrophic lateral sclerosis (ALS) (Saul et al., 2020). However, to the best of our knowledge, there are no reports of BCSFB impairment in VCID patients. Our data revealed a statistically significant reduction in BCSFB K_trans_ in VCID patients compared with healthy controls. It is not clear what causes BCSFB K_trans_ reduction in VCID. Mechanisms that impact tight junctions, or thickening of the choroid plexus walls with age, may, however, be related to the reduction in BCSFB K_trans_ in VCID (Ott et al., 2018; Kant et al., 2018).

As a result of cerebrospinal fluid (CSF) recycling between the subarachnoid space, the brain, and the ventricles, ISF convection is promoted, benefiting both trophic and excretory functions. Fluid clearance occurs across capillaries facilitated by astrocytic endfeet containing aquaporin 4 (AQP-4) and the arachnoid villi are likely to complement CSF reabsorption via multiple pathways (olfactory and spinal arachnoid bulk flow) when CSF pressure and fluid retention are markedly elevated (Johanson et al., 2008). CSF–ISF homeostasis regulates water and solute fluxes at the blood–CSF and blood–brain interfaces. An impaired BBB adversely affects the interstitial environment of neurons (Obermeier et al., 2013). The average CSF turnover rate is between four and five times per day (Telano and Baker, 2023). There is a marked reduction in this rate in AD due to an increase in ventricular space (Ott et al., 2010). CSF turnover decreases with age, this leads to reduced solute clearance, including Aβ (Erickson and Banks, 2013). Interestingly, researchers have demonstrated that senile plague-bearing transgenic mice show a significant impairment in water influx into CSF, similar to the observations made in AQP-4 knockout mice (Igarashi et al., 2014).

However, the role of the BCSFB in determining CSF turnover through the ventricular space has not been fully explored. In this study, we analyzed the data for impairment in movement across the choroid plexus. We used the contrast agent transfer rate into the lateral ventricles as the transfer rate of the CP. Comparing VCID CP transfer rates with those of healthy controls; we observed that in VCID patients contrast agent transfer via the CP was reduced (Figure 2). Both age and pathological conditions change the extracellular matrix components that modulate the extracellular space (ECS) microenvironment and change ECS diffusion (Sykova and Nicholson, 2008; Han et al., 2014; Postnikov et al., 2022; Tonnesen et al., 2023). The hyperintensity acute reperfusion injury marker (HARM), defined as Gd-DTPA enhancement of CSF on fluid-attenuated inversion recovery (FLAIR), was observed in patients with elevated MMP9 (Barr et al., 2010).

The choroid plexus has a unique structure with fenestrated blood vessels and tight junctions located between the ependymal cells forming the surface of the choroid plexus. Removing the choroid plexus reduces CSF production, but continued formation is because of production by cerebral blood vessels. Possibly, our findings can be attributed to the aging process, resulting in a decrease in CSF circulation (Solar et al., 2020).

The BBB functions as a heterogeneous and dynamic barrier (Villabona-Rueda et al., 2019; O’Keeffe et al., 2020), playing an essential role in fluid homeostasis and waste clearance (Sagare et al., 2012; Sengillo et al., 2013). By longitudinally investigating the degree of BBB impairment, this study shows the dynamic nature of the BBB transfer rate in VCID pathology. This observation has potential implications regarding planning treatment strategies based on the brain barriers’ health for patients with cognitive impairment. However, *in vivo* evidence does not fully support the idea that normal aging disrupts the BBB, since current imaging techniques cannot detect subtle BBB changes. The imaging techniques described in the literature are mainly suitable for pathological BBB disruptions.

Considering recent advances in quantitative *in vivo* imaging techniques and the ability to measure inflammation of cerebral barriers noninvasively, quantitative information is now available regarding the focal and distributed effects of diseases on these barriers. Despite this, our study does not answer the following two questions regarding the relationship between barrier impairment and cognitive decline: Does cognitive impairment result from focal impairments of the BBB and BCSFB? And, how does damage to the cerebral barriers affect the onset and course of cognitive impairment?

In this study, we examined both the WM area and the whole brain parenchyma with the exclusion of the CSF compartment for the BBB K_trans_. Notably, the change in BBB K_trans_ in lesion areas may differ because the nature of the lesion may evolve over 2 years. The nature of WM lesion evolution over time is still debatable. Although the Wardlaw group described WM lesions as highly dynamic (Wardlaw et al., 2015), recent studies by Sun et al. (2022) have shown that WMLs are relatively stable over 1–2 years. In future work, separating the BBB K_trans_ in WMLs from that in normal-appearing WM (NAWM) would be more informative. There was no pattern to explain the changes in BBB K_trans_ in either direction. Natural fluctuations in permeability could explain the changes in BBB K_trans_ in either direction, similar to what we observe in MS patients’ brains. The variability of the BBB in our patients limits the usefulness of measurements of BBB permeability in clinical trials of therapeutic agents, since the natural history of BBB changes is too variable to determine the impact of treatment. This is unfortunate since BBB opening is important in the pathophysiology of both AD and VCID.

The results of clustering analysis reveal that using multiple biomarkers provides greater accuracy compared to using one biomarker. Combining the inflammatory biomarker, MMP3, with NAA and K_trans_ provided a complete separation of VCID patients, confirming the importance of a multimodal approach for patient classification. A broad spectrum of biomarkers has been identified that correlate with BBB breakdown such as MMP3 (Candelario-Jalil et al., 2011; Brkic et al., 2015), MMP9 (Barr et al., 2010), PDGF-β (soluble platelet-derived growth factor receptor β) (Payne et al., 2023), VCAM1 (vascular cell adhesion molecule-1) (Haarmann et al., 2015), and ICAM1 (intercellular adhesion molecule 1) (Dietrich, 2002). In this study, we examined MMP3 and MMP9 for their power in VCID classification. We found that MMP3 has greater power for this cohort of VCID classification. Researchers broadly neglect the study of the role of PDGF-β, VCAM1, and ICAM1 in VCID classification, which merits attention.

Employing statistically optimized panels of biomarkers with different origins may help stratify VCID patients into more specific, cohesive groups for individualized treatment plans. Incorporating multiple biomarkers, such as imaging biomarkers, facilitates a more precise diagnosis of the dominant pathogenesis, also provides the opportunity to explore an optimal combination of biomarkers for VCID classification. Despite the increasing use of advanced *in vivo* imaging biomarkers for VCID diagnosis and classification, we need to make additional efforts to develop a gold standard panel of biomarkers that includes imaging biomarkers. For example, consider genotypes associated with cognitive impairment. According to recent studies, the APOE4 mutation has been associated with BBB structural impairment and altered hippocampal BBB K_trans_ (Halliday et al., 2016; Moon et al., 2021). There is also evidence that BBB breakdown contributes to cognitive decline associated with APOE4 (Montagne et al., 2020).

There is a potential limitation in measuring the true passage rate of CSF through the CP. There is a possibility that Gd-DTPA accumulation in the ventricles is not solely related to CP function. Nevertheless, since the cerebral fluid system under investigation is a closed system, we anticipate that multiple flow data, including data on flow through the aqueduct of Sylvius, will provide a reliable biomarker of CP flow. However, in the current comparative study, any change in CP leakage has informative value. Another limitation of the study is the complexity of the experimental methods, involving MR spectroscopy, complex biochemistry, and K_trans_ measurements, which will restrict clinical application. However, diffusion tensor imaging can replace spectroscopy, ultrasensitive fluid measurement instruments can detect inflammation, and cerebrospinal fluid albumin index can measure BBB permeability.

Several studies have demonstrated regional differences in K_trans_ (Ha et al., 2021; Ivanidze et al., 2019). Regional analysis of K_trans_ dynamics would provide more details on regional cerebral barriers’ vulnerability/resilience in VCID. Studying K_trans_ dynamics in anatomical regions relevant to memory and cognition, such as the hippocampus (Montagne et al., 2015; Moon et al., 2021) is of immense value as a continuation of this study.

## 5 Conclusion

Longitudinal *in vivo* studies could significantly increase our knowledge of the pathogenesis variability of VCID classes as well as their evolution. The BBB K_trans_ is dynamic in VCID and inflammatory biomarkers with BBB K_trans_ can be used to separate VCID into distinct groups that suggest different underlying pathologies. The BCSFB K_trans_ showed a decrease in this group of patients with VCID. This highlights the involvement of both CNS barriers in VCID.

## Data Availability

The datasets presented in this study can be found in online repositories. The names of the repository/repositories and accession number(s) can be found in this article/Supplementary material.

## Supplementary material

Supplementary Figure 1 **(A)** The total elevated WM BBB K_trans_ [in mL/(g-min)] was calculated by summing up the voxel values above the K_trans_ threshold in WM. This approach highlights the extend of BBB damage in WM. The same pattern that was observed for the mean BBB K_trans_ in WM is visible here. In this figure, the length of the arrow corresponds to the amount of change in BBB K_trans_, and the blue arrow head indicates the direction in which BBB K_trans_ has changed. The subject with no change in BBB Ktrans is shown only by the arrow head without the length of the arrow. **(B)** As part of the BBB K_trans_ calculation process, an arterial input function (AIF) needed to represent Gd-DTPA concentration ([Gd-DTPA]) in plasma. We used superior sagittal sinus as AIF. This plot shows T1 value that represents [Gd-DTPA] as samples from superior sagittal sinus (SSS) in the first and second rounds of DCE-MRI, respectively. We did not find any statistically significant differences in the AIFs. This finding supports the rigor of data acquisition for DCE-MRI over a two-year period.
