# Supplementary figures and images for: Barriers of the CNS transfer rate dynamics in patients with vascular cognitive impairment and dementia
Source: Front Aging Neurosci. 2024 Sep 25;16:1462302. doi: 10.3389/fnagi.2024.1462302 (PMC11461252; doi:10.3389/fnagi.2024.1462302)

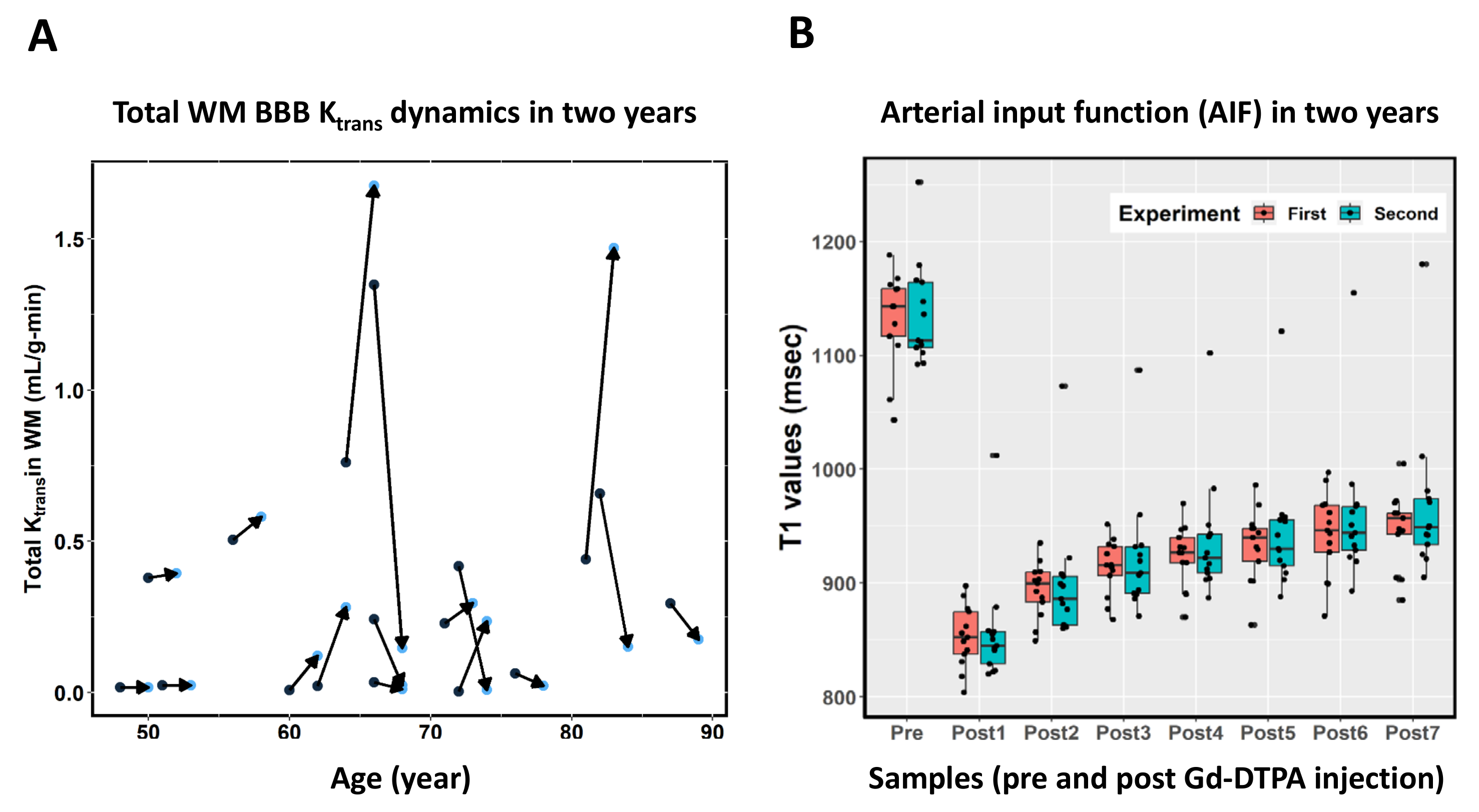

Supplement: Supplementary Figure 1 — (A) The total elevated WM BBB Ktrans [in mL/(g-min)] was calculated by summing up the voxel values above the Ktrans threshold in WM. This approach highlights the extend of BBB damage in WM. The same pattern that was observed for the mean BBB Ktrans in WM is visible here. In this figure, the length of the arrow corresponds to the amount of change in BBB Ktrans, and the blue arrow head indicates the direction in which BBB Ktrans has changed. The subject with no change in BBB Ktrans is shown only by the arrow head without the length of the arrow. (B) As part of the BBB Ktrans calculation process, an arterial input function (AIF) needed to represent Gd-DTPA concentration ([Gd-DTPA]) in plasma. We used superior sagittal sinus as AIF. This plot shows T1 value that represents [Gd-DTPA] as samples from superior sagittal sinus (SSS) in the first and second rounds of DCE-MRI, respectively. We did not find any statistically significant differences in the AIFs. This finding supports the rigor of data acquisition for DCE-MRI over a two-year period. [file Image_1.TIFF]
